# Appendicolith detection in dual-energy CT of adult acute appendicitis: comparing portovenous phase and virtual noncontrast with true noncontrast images

**DOI:** 10.1007/s00261-025-05241-y

**Published:** 2025-11-27

**Authors:** Rathachai Kaewlai, Jitti Chatpuwaphat, Sasima Tongsai, Piyachai Siriphiphatcharoen, Papasorn Wattanakul, Patcharaporn Thaisuriyo, Dhanawin Wongsaengchan, Napakadol Noppakunsomboon, Shanigarn Thiravit

**Affiliations:** 1 Department of Radiology, Faculty of Medicine Siriraj Hospital, Mahidol University, 2 Wanglang Road, Bangkok Noi, 10700 Bangkok, Thailand; 2 Department of Research, Faculty of Medicine Siriraj Hospital, Mahidol University, 2 Wanglang Road, Bangkok Noi, 10700 Bangkok, Thailand; 3 Department of Biochemistry, Faculty of Medicine Siriraj Hospital, Mahidol University, 2 Wanglang Road, Bangkok Noi, 10700 Bangkok, Thailand; 4 Faculty of Medicine Siriraj Hospital, Mahidol University, 2 Wanglang Road, Bangkok Noi, 10700 Bangkok, Thailand; 5 Department of Surgery, Faculty of Medicine Siriraj Hospital, Mahidol University, 2 Wanglang Road, Bangkok Noi, 10700 Bangkok, Thailand

**Keywords:** Appendicitis, Retrospective studies, Tomography, X-ray computed, Appendicoliths, Dual energy

## Abstract

**Objectives:**

Appendicoliths are associated with failed nonoperative management in acute appendicitis and are used to exclude patients from this treatment. This study evaluated whether portovenous phase (PVP) and virtual noncontrast (VNC) images from rapid-kVP-switching dual-energy CT (rsDECT), alone or combined, can reliably detect appendicoliths using true noncontrast (TNC) images as the reference. Additional aims included identifying CT features of overlooked appendicoliths and those linked to complicated appendicitis.

**Methods:**

Consecutive adults with pathologically confirmed appendicitis who underwent preoperative rsDECT and appendectomy were retrospectively included. Two radiologists independently assessed PVP, VNC, PVP + VNC, and TNC images for appendicolith presence and number, with a third resolving discrepancies. Presence was classified as present or absent, and number into 1, 2 and > 2. Agreement with TNC was assessed using kappa statistics. Logistic regression identified predictors of overlooked appendicoliths and features associated with complicated appendicitis.

**Results:**

Among 203 patients; 71 (35%) had appendicoliths. PVP, VNC, and PVP + VNC showed substantial-to-almost-perfect agreement with TNC for detection (kappa = 0.805, 0.793, and 0.817, respectively; all *p* < 0.001) and substantial agreement for numbering (weighted kappa = 0.734, 0.706, and 0.734, respectively; all *p* < 0.001). Overlooked appendicoliths had smaller perimeters (OR 12.303; *p* = 0.003) and lower attenuation (OR 10.456; *p* = 0.004). In cases with appendicoliths, larger minimum length predicted complicated appendicitis (OR 16.756; *p* = 0.013).

**Conclusions:**

PVP and VNC from rsDECT sufficiently detected and numbered appendicoliths. Small, low attenuation appendicoliths were easily overlooked, while larger ones were linked to complications.

**Supplementary Information:**

The online version contains supplementary material available at 10.1007/s00261-025-05241-y.

## Introduction

Identifying appendicolith or calcific foci in patients with acute appendicitis has garnered increasing interest, as their presence is considered a contraindication for nonoperative management (NOM) of acute appendicitis in adults, according to the World Society of Emergency Surgery (WSES) guidelines [1]. This recommendation is supported by several studies-including a few prospective ones-demonstrating a strong association between appendicoliths and complications such as gangrene or perforation [2, 3, 4].

Computed tomography (CT) is the standard imaging modality for evaluating suspected appendicitis in adults [5]. Beyond diagnosis, CT is well-suited to detect appendicoliths due to their internal calcification [6, 7], as CT is widely accepted for identifying calcifications throughout the body [8, 9]. Detection is more accurate on noncontrast images, as contrast enhancement can obscure calcific foci by increasing adjacent soft tissues attenuation based on our experience [6]. However, most abdominal CTs for suspected appendicitis are performed with intravenous contrast medium in the portovenous phase (PVP), omitting noncontrast images to reduce radiation. This may lead to overlooked appendicoliths; for instance, 11.8% were overlooked on contrast-enhanced scans compared to noncontrast images [6]. Dual-energy CT (DECT) may offer a solution as it acquires datasets at two different energy spectra, allowing for material decomposition and generation of virtual noncontrast (VNC) images from PVP scans without additional radiation, which is especially beneficial in younger patients. There are three main DECT configurations in current practice: dual source, rapid kVp switching, and dual-layer detector systems. Each has unique strengths and limitations, but all allow creation of VNC images that may serve as substitutes for true noncontrast (TNC) scans. Prior research in urinary calculi shows VNC has moderate sensitivity compared to TNC images [10, 11], though its effectiveness for detecting appendicolith is uncertain. This study aimed to compare the detection of appendicoliths on PVP, VNC, and combined PVP + VNC images, using TNC images as the reference standard, in patients with pathologically confirmed acute appendicitis.

## Materials and methods

### Study design and subjects

**Fig. 1:**
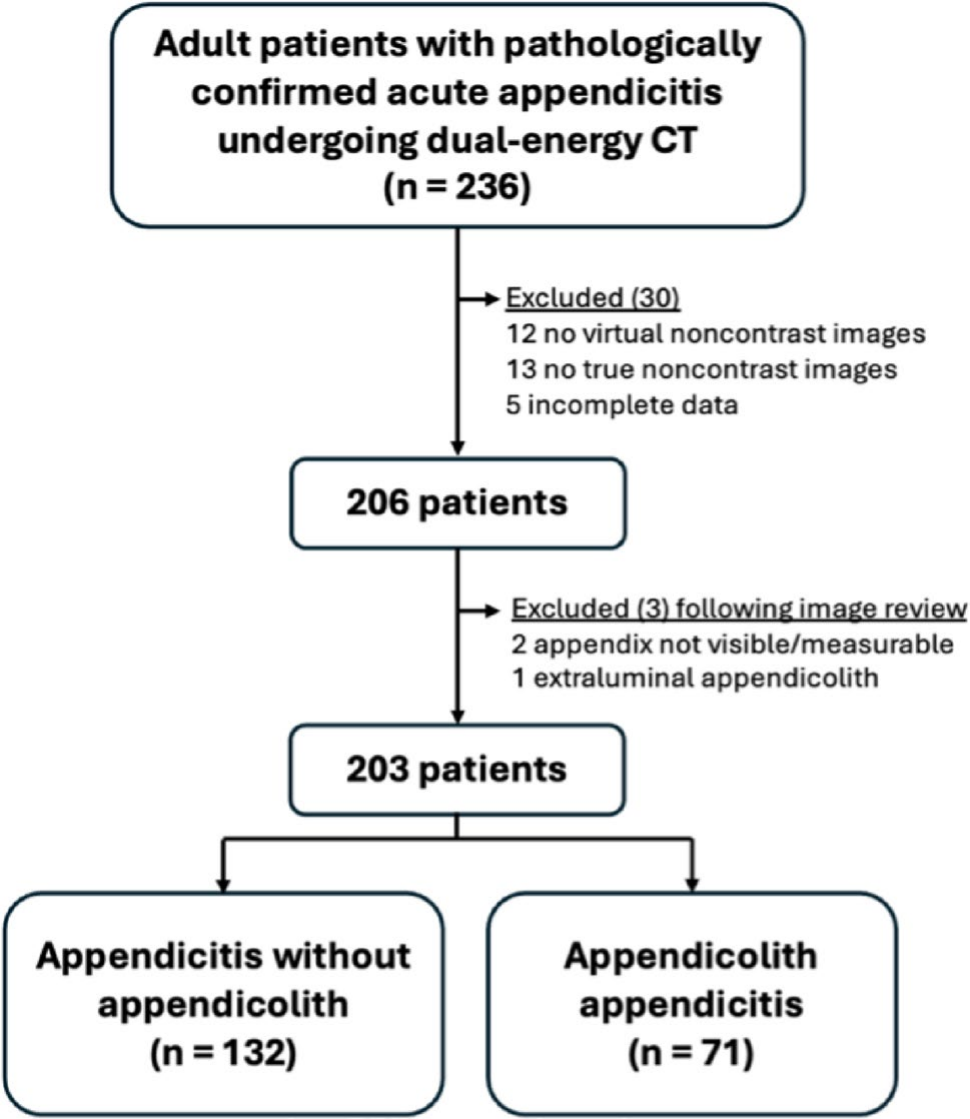
Patient inclusion chart. *TNC* true noncontrast, *VNC* virtual noncontrast

This retrospective, analytic, cross-sectional investigation was conducted at a 2200-bed, urban medical center with approval from the hospital’s Institutional Review Board (certificate of approval number Si 041/2025). Informed consent was waived due to the retrospective nature of the study and the minimal associated risks. A total of 203 CT scans from 203 consecutive adult patients aged 18 years or older were included, identified through a hospital database search of International Classification of Diseases, 10th revision (ICD-10) codes related to acute appendicitis and appendectomy between February 2020 and December 2024 with preoperative DECT (*n* = 236). The studies without true noncontrast (TNC) imaging (*n* = 13), virtual noncontrast (VNC) images (*n* = 12), and incomplete data (*n* = 5) were excluded. After a re-review by radiologists, three additional studies were excluded because of nonidentifiable appendix (*n* = 2) and extraluminal appendicolith (*n* = 1). The remaining examinations (*n* = 203) were included for data collection and analysis (Fig. 1). Of these, 134 patients overlapped with a previously reported cohort evaluating low-keV images [12], and 17 with a cohort assessing the association between appendicoliths and complicated appendicitis [6]. During the same period, 1,260 single-energy CT scans were performed in adult patients with pathologically confirmed acute appendicitis.

### DECT acquisition

All DECT studies were conducted using one of three single-source, rapid-kVp switching DECT scanners (Revolution CT or Revolution Apex, GE Healthcare). Each examination included both a true noncontrast (TNC) phase and a contrast-enhanced portovenous phase (PVP). TNC scans were acquired using the following parameters: fast kV switching, tube current of 145 mA, helical pitch of 0.992:1, and scan rotation time of 0.8 s. Images spanned from the diaphragmatic domes or renal upper poles to the level of ischial tuberosities. The PVP images were obtained approximately 70–80 s after intravenous contrast injection with scanning parameters consisting of a tube voltage alternating between 80 and 140 kVp, tube current of 280 mA, a helical pitch of 0.992:1, and a gantry rotation time of 0.6 s. Intravenous contrast material was delivered via an automatic injector at a rate of 2–3 mL/s, with a total volume based on patient weight (1.5-2.0 mL/kg). Postprocessing was performed on a dedicated GE Advantage Workstation (AW 4.7), generating VNC images using iodine suppression algorithms, as well as 70 monoenergetic images as a surrogate for standard 120-kVp-equivalent PVP images. All datasets were reconstructed at 1.25-mm slice thickness and archived in the Picture Archiving and Communication System (PACS) for subsequent interpretation. A random sample of 40 studies was collected for radiation dose information. For both non-contrast and portovenous phases, the mean volume CT dose index (CTDIvol) was 8.54 mGy (SD: 1.21), while the mean dose length product (DLP) was 803 mGy-cm (SD: 176.28). If the non-contrast phase had not been performed, the DLP would have been approximately 47.1% of these values (SD: 5.1).

### Image re-interpretation

Two radiologists-one abdominal radiologist with 14 years of experience and one emergency radiologist with 9 years of experience-independently re-reviewed all DECT examinations. They were blinded to clinical data aside from the diagnosis of appendicitis. Re-interpretation was conducted in two separate sessions, six weeks apart. In the first session, the radiologists reviewed the PVP images followed by the VNC images, documenting the presence and number of appendicoliths. In the second session, they assessed the TNC images for the presence, number, and type of appendicoliths. For each session, the radiologists decided whether appendicoliths were present or absent. If more than one appendicolith was present, the largest one (“representative appendicolith”) was selected for characterization. The representative appendicoliths were classified into two types, as described in a previous study [6]: type 0 (rounded and homogeneous appearance) and type 1 (oval, and ring-like or heterogeneous appearance). Other included characteristics were location, presence of obstruction, size and attenuation parameters. Discrepancies between the two primary readers were resolved by a third radiologist, an emergency radiologist with 24 years of experience. This reviewer also documented additional imaging features of the representative appendicoliths and appendix diameter on CT.

### Reference standards

The diagnosis of acute appendicitis was confirmed via histopathological analysis. Complicated appendicitis was defined on operative and histopathological findings, with perforation and gangrene defined either on operative or histopathological diagnoses. Appendicoliths were considered present when identified on TNC images, as determined by three radiologists using the above methods. They were defined as foci of at least 2 mm in size with attenuation greater than that of surrounding appendiceal structures or nearby muscle tissue [7, 13]. Complicated appendicitis was defined as gangrenous or perforated appendicitis [14].

### Statistical analysis

Categorical variables, including sex, presenting symptoms, physical signs, and CT findings, were expressed as frequencies and percentages. Continuous variables, such as age, body mass index, temperature, time from presentation to CT, and various duration metrics, were summarized as mean ± standard deviation (SD) when normally distributed or as median and range when distributions were non-normal.

Comparative analyses were conducted between patients with and without appendicolith. For categorical data, comparisons utilized either Yates’ continuity-corrected chi-square test or Fisher’s exact test, as appropriate. Continuous variables were assessed using independent samples t-test for normally distributed data, and Mann-Whitney *U* test for non-normally distributed data. Univariable logistic regression was first applied to explore potential predictors of appendicolith presence. Variables with p-values below a predefined threshold were included in a forward stepwise multivariable logistic regression model to identify independent predictors. Results were presented as odd ratios (ORs) with corresponding 95% confidence intervals (CIs).

Inter-reader agreement among the PVP, VNC and PVP + VNC series compared with the reference TNC for detecting appendicoliths was evaluated using Cohen’s kappa (κ) statistics. Dichotomous agreement (presence vs. absence of appendicoliths) was assessed with unweighted κ, whereas agreement on a three-tiered ordinal scale (e.g., 0, 1, *≥* 2 appendicoliths) was assessed with weighted κ using quadratic weights. For each κ statistics, 95% confidence interval (CI), and *p*-value were provided. The 95% CIs were derived from the asymptotic standard error (ASE) provided in the SPSS output, using the formula κ ± 1.96 × ASE. The strength of agreement was interpreted according to the benchmarks proposed by Landis and Koch (1977) [15].

Logistic regression analyses were conducted to determine imaging predictors of overlooked appendicoliths on PVP + VNC, with results presented as odds ratios (ORs) and 95% CIs. To enhance clinical interpretability, receiver operating characteristics (ROC) curve analyses were conducted to determine optimal cutoff values for significant continuous predictors (appendicolith perimeter and attenuation). For each cutoff, the area under the ROC curve (AUC), sensitivity, and specificity were reported. Subgroup analyses were performed among patients with appendicolith appendicitis to identify CT findings associated with complicated appendicitis.

All statistical analyses were performed using IBM SPSS for Windows, version 27. A two-tailed *p*-value of < 0.05 was considered statistically significant throughout.

## Results

The included patients had a mean age of 50.0+/- 18.6 years, with 38.4% male, and a median Alvarado score of 6. Of these, 124 cases (124/203; 61.1%) were classified as uncomplicated appendicitis based on surgical and histopathological results. Baseline patient characteristics are summarized in Table 1, and predictive factors for appendicolith appendicitis are listed in Table 2. Univariable and multivariable analyses identified two factors significantly associated with appendicolith appendicitis: median maximum diameter of appendix (adjusted OR 1.176; 95% CI 1.053–1.314; *p* = 0.004) and the presence of complication (adjusted OR 2.645; 95% CI 1.331–5.255; *p* = 0.005).

**Fig. 2:**
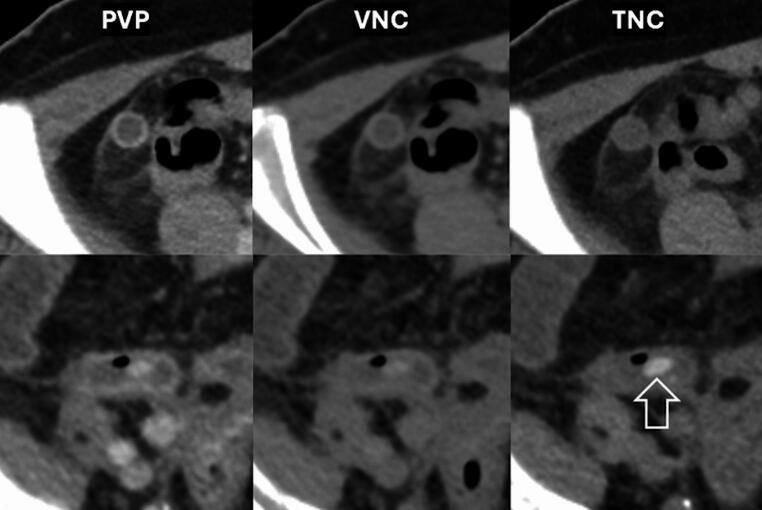
Example of cases with clear absence (top row) and presence (bottom row) of appendicoliths on portovenous phase (PVP), virtual noncontrast (VNC), and true noncontrast (TNC) dual-energy CT. Arrow = appendicolith

There was substantial-to-almost-perfect agreement between PVP, VNC, and PVP + VNC images in identifying appendicoliths compared with TNC, which served as the reference standard, with kappa values (95% CI) of 0.805 (0.716, 0.894), 0.793 (0.702, 0.884), and 0.817 (0.731, 0.903), respectively (**Supplementary Table 1**). For numbering appendicoliths, the three-category classification comparison showed substantial agreement between PVP, VNC, PVP + VNC, with TNC as the reference standard, with weighted kappa values (95% CI) of 0.734 (0.574, 0.894), 0.706 (0.543, 0.869) and 0.734 (0.575, 0.894), respectively (**Supplementary Table 2**). Figure 2 demonstrates cases with agreement among three image series.

**Table 1:** Comparison of patients’ demographics, clinical characteristics and appendix diameter between appendicitis without and with appendicolith (*n* = 203)

Factors	All patients (*n* = 203)	Appendicitis without appendicolith (*n* = 132)	Appendicitis with appendicolith (*n* = 71)	*P* values
Mean age (SD)	50.0 (18.6)	48.5 (18.6)	52.7 (18.4)	0.127
Male sex (%)	78 (38.4)	54 (40.9)	24 (33.8)	0.400
Mean body mass index (SD)	23.8 (4.5)	23.9 (4.8)	23.6 (4.0)	0.635
Median durations				
From symptoms to arrival (hr; range) (*n* = 199)	24 (1, 240)	24 (1, 240)	24 (1, 168)	0.798
From symptoms to CT (hr; range) (*n* = 199)	5.5 (0.3, 43.1)	5.5 (0.3, 27.5)	5.8 (1.2, 43.1)	0.745
Symptoms and signs				
Right lower quadrant pain (%) (*n* = 200)	191 (95.5)	126 (96.9)	65 (92.9)	0.282
Mean body temperature (^0^C; *n* = 198)	37.1 (0.7)	37.0 (0.6)	37.2 (0.9)	0.100
Rebound tenderness (*n* = 200)	101 (50.5)	62 (47.7)	39 (55.7)	0.350
Migratory pain (*n* = 200)	102 (51.0)	71 (54.6)	31 (44.3)	0.184
Anorexia (*n* = 200)	72 (36.0)	43 (33.1)	29 (41.4)	0.308
Nausea and vomiting (*n* = 200)	103 (51.5)	63 (48.5)	40 (57.1)	0.306
Laboratory results				
Median white blood cell counts (mm^3^; range)	12,900 (10, 28490)	13,220 (10, 28490)	12,460 (1030, 25060)	0.268
% median neutrophil (range)	83.0 (0, 97)	83 (0, 93.9)	81.9 (12.4, 97)	0.741
Median absolute neutrophils	10,538 (0, 24769.4)	11,165 (0, 24769.4)	9794.4 (127.7, 22554)	0.280
% median lymphocyte (range) (*n* = 191)	10.8 (1.9, 47.5)	10.2 (1.9, 47.5)	11.2 (2.0, 42.0)	0.755
Neutrophil-lymphocyte ratio (*n* = 191)	7.9 (0.4, 40.1)	8.1 (0.4, 40.1)	7.4 (1.1, 37.3)	0.620
Median Alvarado score (*n* = 201)	6 (0, 10)	6 (2, 10)	6 (0, 10)	0.738
Median maximum diameter of appendix (mm; range)	12.5 (6.2, 27.4)	11.8 (6.2, 22.0)	13.8 (7.5, 27.4)	*< 0.001*
Categories of appendicitis Uncomplicated Complicated	124 (61.1) 79 (38.9)	96 (72.7) 36 (27.3)	28 (39.4) 43 (60.6)	*< 0.001*
Treatment (*n* = 202)				0.712
Open appendectomy	158 (78.2)	104 (79.4)	54 (76.1)	
Laparoscopic appendectomy	44 (21.8)	27 (20.6)	17 (23.9)	

**Table 2:** Univariable and multivariable logistic regression analyses of predictive factors of having appendicolith appendicitis (*n* = 71)

	Univariable model		Multivariable model	
Factors	Unadjusted OR (95% CI)	*P* Value	Adjusted OR (95% CI)	*P* Value
Body temperature	1.495 (0.974, 2.294)	0.066	-	-
Appendix diameter (mm)	1.225 (1.113, 1.348)	*< 0.001*	1.176 (1.053, 1.314)	*0.004*
Complicated appendicitis	4.095 (2.223, 7.544)	*< 0.001*	2.645 (1.331, 5.255)	*0.005*

**Fig. 3:**
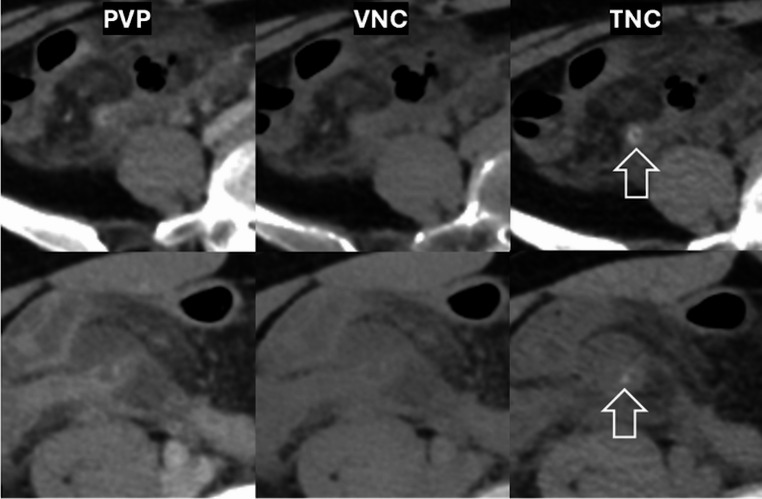
Example of two cases with difficult detection of appendicolith on portovenous phase (PVP) and virtual noncontrast (VNC) dual-energy CT. In the top row, an appendicolith was rather large and clearly hyperattenuating on TNC but it was difficult to appreciate on PVP and VNC. In the bottom row, small size and low attenuation likely make the appendicolith difficult to detect on PVP and VNC. *Arrow* appendicolith

Several CT features of appendicoliths differed significantly between overlooked appendicoliths and those detected on PVP + VNC images in the univariable analysis, including the presence of obstruction, all size parameters, and certain attenuation parameters (Table 3). In multivariable analysis, only two factors remained independently predictive of nonvisualization on PVP + VNC: smaller perimeter (adjusted OR, 12.303; 95% CI, 2.377, 63.690, *p* = 0.003) and lower average attenuation (adjusted OR, 10.456; 95% CI, 2.095, 52.191; *p* = 0.004) (Table 4). Cutoff thresholds are presented in Table 4 footnotes. Figure 3 shows cases with overlooked appendicoliths.

**Table 3:** Comparison of CT characteristics of appendicoliths that were overlooked on a combination of portovenous-phase (PVP) and virtual Noncontrast (VNC) CT, and those that were detected (*n* = 71)

	All patients (*n* = 71)	Overlooked appendicolith on PVP + VNC (*n* = 19)	Detected appendicolith (*n* = 52)	*P* values
Type 0 (n, %)	50 (70.4)	14 (73.7)	36 (69.2)	0.944
Location (n, %)				0.616
Proximal	33 (46.5)	7 (36.8)	26 (50.0)	
Mid	16 (22.5)	5 (26.3)	11 (21.2)	
Distal	22 (31.0)	7 (36.8)	22 (28.8)	
Obstruction (n, %)	28 (39.4)	3 (15.8)	25 (48.1)	*0.029*
Size parameters (median, range)				
Minimum diameter (mm)	4.9 (0.9, 13.8)	3.4 (1.8, 11.5)	5.7 (0.9, 13.8)	*0.013*
Maximum diameter (mm)	7.0 (1.8, 20.2)	4.2 (2.5, 14.4)	7.5 (1.8, 20.2)	*0.006*
Area (cm^2^, range)	0.23 (0.02, 1.68)	0.11 (0.03, 1.29)	0.29 (0.02, 1.68)	*0.004*
Perimeter (cm; range)	2.02 (0.55, 5.35)	1.28 (0.79, 4.07)	2.30 (0.55, 5.35)	*0.004*
Attenuation parameters (HU)(median, range)				
Average attenuation	119.0 (58.6, 490.0)	91.1 (58.6, 127.0)	132.0 (64.0, 490.0)	*< 0.001*
Minimum attenuation	55.0 (-258.0, 162.0)	50.0 (-258.0, 87.0)	56.0 (-219.0, 162.0)	0.455
Maximum attenuation	186.0 (20.6, 907.0)	139.0 (91.0, 190.0)	217.5 (20.6, 907.0)	*< 0.001*
Standard deviation of attenuation	39.5 (13.6,246.0)	32.5 (18.4, 72.1)	41.7 (13.6, 246.0)	0.245
Difference between average attenuation of appendicolith and surrounding tissues	88.6 (12.0, 452.3)	62.2 (16.5, 166.1)	104.5 (12.0, 452.3)	*< 0.001*

^a^ if there was more than one appendicolith, only the representative one was measured

**Table 4:** Univariable and multivariable logistic regression analyses of predictive factors of nondefinitive diagnosis of appendicoliths on a combination of portovenous-phase (PVP) and virtual Noncontrast (VNC) CT (*n* = 71)

	Univariable model		Multivariable model	
Factors	Unadjusted OR (95% CI)	*P* Value	Adjusted OR (95% CI)	*P* Value
Perimeter ≤ 2 mm^1^	4.480 (1.399, 14.348)	*0.012*	12.303 (2.377, 63.690)	*0.003*
Average attenuation ≤ 90 HU^2^	4.950 (1.530, 16.014)	*0.008*	10.456 (2.095, 52.191)	*0.004*

¹ ROC analysis: AUC = 0.724 (95% CI: 0.584, 0.864, *p* = 0.004); cutoff of perimeter ≤ 2 mm, sensitivity 73.7%, specificity 61.5%

² ROC analysis: AUC = 0.811 (95% CI: 0.714, 0.909, *p* < 0.001); cutoff of average attenuation ≤ 90 HU, sensitivity 47.4%, specificity 84.6%

Four CT features of appendicoliths were associated with complicated appendicitis in univariable analysis: appendicolith obstruction, larger perimeter, and longer maximum and minimum lengths. However, only one factor remained independently predictive of complication in multivariable analysis-the minimum length of appendicoliths (OR 16.756) (Table 5). A cutoff of 5 mm for the minimum length showed 59.5% sensitivity and 71.4% specificity for predicting complicated appendicitis.

**Table 5:** Univariable and multivariable logistic regression analyses of appendicolith features predictive of complicated appendicitis (*n* = 71)

	Univariable model		Multivariable model	
Factors	Unadjusted OR (95% CI)	*P* Value	Adjusted OR (95% CI)	*P* Value
Obstructed appendicolith	2.864 (1.008, 8.132)	*0.048*	-	-
Perimeter (cm) of appendicolith	1.568 (0.971, 2.535)	0.066	-	-
Maximum length (cm) of appendicolith	3.275 (0.837, 12.821)	0.088	-	-
Minimum length (cm)^1^ of appendicolith	16.756 (1.967, 142.768)	0*.010*	16.756 (1.967, 142.768)	*0.010*

^1^Area under the ROC curve = 0.695 (95% CI: 0.571, 0.818, *p* = 0.006)

At a cutoff appendicolith diameter of 0.5 cm, the OR was 3.676 (95% CI: 1.318, 10.253; *p* = 0.013), while the sensitivity and specificity was 59.5% and 71.4%, respectively, in predicting complicated appendicitis

## Discussion

In this study of 203 patients with appendicitis who underwent preoperative rsDECT, we found that PVP, VNC, and combined PVP + VNC images were generally sufficient for detecting and numbering appendicoliths, suggesting that TNC acquisition may not always be necessary. Some appendicoliths were overlooked on PVP and VNC images, often being small or lower in attenuation; the clinical significance of missing these smaller appendicoliths remains uncertain. Notably, larger appendicoliths were significantly associated with complicated appendicitis, underscoring the importance of careful interpretation. Eliminating routine TNC acquisition could substantially reduce radiation exposure, a particularly meaningful consideration in the predominantly younger appendicitis population.

Among our cohort, 71 out of 203 patients (35%) had appendicoliths, aligning with prior CT-based studies reporting rates from 25.5% to 47.1% [7, 13, 17, 18]. Some reports suggest CT underestimates prevalence compared to pathology [19–22]. While CT may not detect every appendicolith, it remains crucial in appendicitis management, highlighting the importance of accurate preoperative imaging. Our findings confirmed the association between appendicoliths and complicated appendicitis, as reported in several studies [6, 13, 16, 17, 18, 20–33], though conflicting results have also been reported [34–37].

We observed substantial-to-almost-perfect agreement between PVP, VNC, and PVP + VNC with TNC in detecting and counting appendicoliths with kappa values comparable to Kim HY et al. [38]. Prior studies, such as by Mariadason et al. [19], have shown variable inter-reader agreement (kappa 0.48–0.83), underscoring diagnostic challenges in appendicolith detection. Incorporating VNC as part of DECT may help improve agreement, as calcifications can theoretically be easier to detect on these images. Although we did not formally assess observer agreement, we note that accurate detection of appendicoliths on PVP requires recognizing their higher attenuation compared to muscle-not the inflamed appendix wall-which can be subtle (Fig. 2). While VNC did not outperform PVP in detection or numbering in our study, this was not the primary research question and our sample size was limited. Nonetheless, we consider VNC a valuable component of DECT for calcification detection, with trade-offs mainly in reader time and visual fatigue, rather than radiation, as no additional scan is required.

Appendicoliths overlooked on PVP + VNC were smaller and lower in attenuation (Fig. 3), reducing conspicuity. While VNC theoretically improves visibility of dense foci, it may reduce calcification size and attenuation, impairing detection. Cheng Y et al. [39] found urinary calculi appeared smaller on VNC than on lower-keV images. A meta-analysis reported pooled sensitivity of only 78.1% for DECT (mostly dual-source, dual-energy platform) in detecting urinary stones [10], attributing misses to thicker slices (> 2 mm). The rsDECT platform which uses two-material decomposition, is known to have lower tissue differentiation than dual-source systems [40]. Bone windows may aid detection [41], but small, low-density stones remain challenging. These overlooked stones may be clinically insignificant, as larger size was independently associated with complications in our study-consistent with prior reports [13, 18, 20, 33]. Proposed size cutoff varies widely, from 2.48 mm [20] to 10 mm [13]. In our analysis, a 5-mm cutoff offered reasonable specificity but low sensitivity. Other reported predictors such as base location [18] or obstruction [42] were not significant in our cohort. We did not evaluate mural enhancement, which has been suggested as a predictor [18].

Our study has limitations. First, it is a retrospective single-center study, selection bias may be present. Second, although TNC was used as the reference standard, pathology may detect more appendicoliths [19–22]. We did not have consistent surgical or pathological documentation, as appendicoliths are not routinely recorded in operative or pathology reports at our hospital. Therefore, we relied on radiologist consensus on TNC as the reference standard, acknowledging this is imperfect and may underestimate the true prevalence of appendicoliths. For this reason, we chose to evaluate diagnostic agreement with Cohen’s kappa statistics instead of performance analyses. Third, although two radiologists independently reviewed all examinations, discrepancies were adjudicated by a third senior radiologist to ensure consistent final interpretations. While this consensus process enhanced reliability and clinical applicability, it precluded formal inter-reader agreement analysis. Fourth, most patients still received TNC because scan protocols were selected according to individual radiologists’ preferences, and baseline familiarity with TNC was high in our department. Fifth, our findings are specific to rsDECT platform and may not generalize to other technologies. Although all three major DECT configurations can generate VNC images, the accuracy and quality of material decomposition may differ between platforms, and their performance in reliably depicting appendicoliths remains to be evaluated. Sixth, our sample size limited subgroup analyses for rare features. Seventh, this study did not aim to assess the utility of DECT over single-energy CT (SECT), as addressing that question would require a much larger sample size and different evaluation methods. Such studies would be valuable, as they could clarify whether shifting from SECT to DECT provides substantial benefits, particularly given that many current practices still rely on SECT as the primary workhorse modality. Lastly, since appendicoliths are subtler on PVP than TNC, radiologists should interpret PVP images carefully, especially in the absence of other signs of complicated appendicitis.

In conclusion, PVP and VNC from rsDECT reliably detected and numbered appendicoliths, which may obviate the need for TNC scans. Eliminating TNC could also reduce radiation by over 50%, a meaningful advantage given that appendicitis often affects younger patients. Small, low attenuation appendicoliths were sometimes overlooked; although larger appendicoliths were independently linked to complicated appendicitis, the clinical significance of missed smaller appendicoliths remains uncertain due to lack of surgical or pathological outcome data.

## Supplementary Information

Below is the link to the electronic supplementary material.

Supplementary Material 1

## Data Availability

No datasets were generated or analyzed during in this study.

## Abbreviations

CT: Computed tomography
DECT: Dual-energy CT
PACS: Picture Archiving and Communication System
PVP: Portovenous phase
RIS: Radiological Information System
rsDECT: Rapid-kVp switching dual-energy CT
TNC: True noncontrast
VNC: Virtual noncontrast
