# Supplementary material for: Appendicolith detection in dual-energy CT of adult acute appendicitis: comparing portovenous phase and virtual noncontrast with true noncontrast images
Source: Abdom Radiol (NY). 2025 Nov 27;51(6):2776–84. doi: 10.1007/s00261-025-05241-y (PMC13109209; doi:10.1007/s00261-025-05241-y)
Supplement: Supplementary file 1 — Supplementary Material 1 [file 261_2025_5241_MOESM1_ESM.docx]

**Supplementary Table 1.**

**Inter-reader agreement for the presence or absence of appendicoliths compared with TNC (unweighted Cohen’s kappa).**

| **Comparison** | **Kappa (κ)** | **95% CI** | **P value** | **Interpretation^*^** |
| --- | --- | --- | --- | --- |
| PVP vs TNC | 0.805 | 0.716 – 0.894 | < 0.001 | Substantial–Almost perfect |
| VNC vs TNC | 0.793 | 0.702 – 0.884 | < 0.001 | Substantial–Almost perfect |
| PVP+VNC vs TNC | 0.817 | 0.731 – 0.903 | < 0.001 | Almost perfect |

95% confidence intervals for κ were computed from the asymptotic standard error (ASE) reported by SPSS using κ ± 1.96×ASE.

**Supplementary Table 2.**

**Inter-reader agreement for the number of appendicoliths (0, 1, ≥2) compared with TNC (quadratic weighted Cohen’s kappa).**

| **Comparison** | **Weighted kappa (κ)** | **95% CI** | **P value** | **Interpretation^*^** |
| --- | --- | --- | --- | --- |
| PVP vs TNC | 0.734 | 0.574 – 0.894 | < 0.001 | Substantial |
| VNC vs TNC | 0.706 | 0.543 – 0.869 | < 0.001 | Substantial |
| PVP+VNC vs TNC | 0.734 | 0.575 – 0.894 | < 0.001 | Substantial |

^*^Interpretation of κ values based on Landis & Koch (1977) [Reference #15]:

- 0.61–0.80 = *Substantial agreement*
- 0.81–1.00 = *Almost perfect agreement*

P values are two-sided; values < 0.001 are reported as p < 0.001.
